# Coding complete genome of LPAI H16N3 virus from Australia suggests intercontinental movement

**DOI:** 10.1128/mra.01437-25

**Published:** 2026-05-28

**Authors:** Nicole Brook, Simon Hollamby, Matthew Neave, Vittoria Stevens, Michelle Wille

**Affiliations:** 1 Department of Primary Industries and Region Development, Perth, Australia; 2 CSIRO Australian Centre for Disease Preparedness 64808 , Geelong, Victoria, Australia; 3 WHO Collaborating Centre for Reference and Research on Influenza, at the Peter Doherty Institute for Reference and Research on Influenza, Melbourne, Victoria, Australia; 4 Department of Microbiology and Immunology, University of Melbourne, at the Peter Doherty Institute for Reference and Research on Influenza 534133 , Melbourne, Victoria, Australia; Katholieke Universiteit Leuven, Leuven, Belgium

**Keywords:** avian influenza, low pathogenicity avian influenza, H16, gulls, disease ecology, surveillance studies

## Abstract

Herein, we report on the second low pathogenicity avian influenza (LPAI) H16N3 genome sequenced in Oceania. Revealing the viral diversity in Australia’s wild bird reservoir, including in gulls, is critical to the preparation for the arrival of HPAI H5N1.

## ANNOUNCEMENT

Surveillance for avian influenza viruses (AIV) (species: *Alphainfluenzavirus influenzae*, Family: *Orthomyxoviridae*) in wild birds is crucial to better understand the ecology and evolution of these viruses and continue to confirm the absence of high-pathogenicity avian influenza (HPAI) H5N1. Herein, fecal environmental samples were collected as part of the National Avian Influenza Wild Bird program (1) in November 2024 at Coodanup foreshore (32°33′45.0″S 115°44′43.9″E) in the Peel Harvey estuary, a roost site for shorebirds. As fecal environmental samples were collected, no animal ethics was required. During the time of sampling, 50–60 Silver Gulls (*Chroicocephalus novaehollandiae*) were present, in addition to Pacific Black Ducks (*Anas superciliosa*).

Fecal swabs were placed in virus transport media, assayed for AIV, and sequenced using established and accredited approaches, as outlined in references 2, 3. Only a single sample was positive in an AIV matrix qPCR assay (4) with a Ct < 32 and submitted for sequencing. Gene segments were reverse-transcribed and amplified using universal primers (5), and dual index libraries were prepared using a Nextera XT DNA Library Preparation Kit and sequenced on an Illumina MiSeq using a 300 cycle V2 Reagent Kit (2 × 150 paired end). The total reads obtained were 474,660. Sequence reads were trimmed for quality (BBDuk trimmer http://sourceforge.net/projects/bbmap/) and mapped (Geneious mapper, five iterations) against Australian reference strains (including H1-16, N1-9) using Geneious Prime 2023 (Biomatters) (Table 1) (2). Herein, a coding complete sequence was generated for all eight segments of an H16N3: A/wild waterbird/Western Australia/AS-24-3244-0017/2024(H16N3) (Table 1).

**TABLE 1:** Coverage statistics for A/wild waterbird/Western Australia/AS-24-3244-0017/2024(H16N3)

Segment	No. of reads	Average depth	Min depth	Max depth	Segment length (bp)	GC (%)	GenBank accession
1 PB2	31,329	1,968	191	3,722	2,280	44.3	PX588658
2 PB1	44,487	2,782	113	8,421	2,274	42.6	PX588659
3 PA	913	59	10	121	2,151	43.8	PX588660
4 HA-H16	21,742	1,832	158	3,292	1,698	40.8	PX588661
5 NP	66,798	6,370	1,490	9,697	1,497	47	PX588662
6 NA-N3	84,565	8,845	739	15,981	1,410	43.8	PX588663
7 MP	120,500	17,658	3,395	24,047	982	46.6	PX588664
8 NS	85,036	14,382	3,293	20,094	838	44.6	PX588665

**Fig 1:**
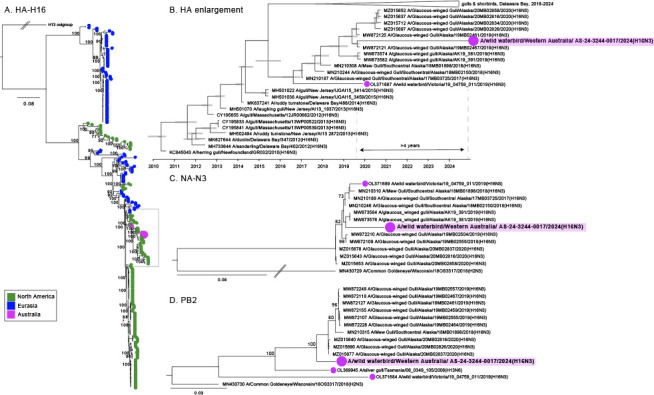
(**A**) Maximum likelihood tree of all H16-HA sequences in GenBank. Tips are colored by geographic region. Smaller purple circles comprise previously sequenced H13 and H16 genomes from Australia, and the large purple circle corresponds to the genome sequenced in this study. (**B**) Time-scaled phylogeny of the clade of H16 sequences identified within a box in A. (**C, D**) Maximum likelihood tree of the top 10 blast hits and Australian H16 (and H13 for PB2) viral sequences for the (**C**) N3-NA and (**D**) PB2 segment. For (**A**, **C**, and **D**), the scale bar corresponds to the number of nucleotide substitutions per site, and ultrafast bootstrap values are provided for major nodes. For (**B**), the scale bar indicates time in years, and node bars correspond to the 95% highest posterior density of node height. Phylogenetic analysis was undertaken as per references (2, 3). In all cases, sequence segment nucleotide sequences were aligned using MAFFT (6) integrated in Geneious Prime. Maximum likelihood trees were constructed using iq-tree2 (7) with the best-fit substitution model. Time-structured trees were constructed using BEAST v1.10.4 (9) with parameters as per references (2, 3).

The H16N3 generated is the second genome of this subtype sequenced in Oceania. Against expectation, the viral genome was not highly similar to the previously reported H16 (HA 96%, NA 95.4%, internals 81–92% with the exception of M, which is 97.4%), to A/wild bird/Victoria/19-04759-011/2019 (H16N3), or H13 (internals 85–92% similar to A/Silver Gull/Tasmania/06-0349-105/2006 (H13N6) sequences from Australia. Rather, the top blast hits (*n* = 20) and phylogenetic analysis (using MAFFT, iq-tree2 [6, 7]) reveal that the H16 virus sequenced here was consistently most similar to sequences from gulls sampled in Alaska in 2019 and 2020 (>98% percentage identity), including both the glycoproteins (HA, NA, Fig. 1B and C) and internal segments (e.g., PB2; Fig. 1D). Time-structured analysis (using MAFFT [6], TempEst [8], BEAST v1.10.4 [9] with parameters as per references 2, 3) reveals a long branch length between sample collection and the mean most recent common ancestor (MRCA) of the closest blast hits (>4 years) (Fig. 1B). This suggests circulation in a population that has not previously been sampled (sampling of gulls is not routinely undertaken in most of Asia or Australia). While gulls are the major reservoir for H13 and H16 viruses (10), Australian gull species are endemic or restricted to the southern hemisphere (11), such that the mechanism of viral incursion into Australia is unclear. Reassortment between HPAI H5N1 and LPAI viruses from gulls likely contributed substantially to HPAI H5N1 outbreaks in seabirds in 2022 (12, 13), such that revealing the viral diversity in Australia’s wild bird reservoir, including in gulls, is critical to preparation for the arrival of HPAI H5N1.

## Data Availability

The full genome has been deposited in GenBank (accession PX588658–PX588665), and raw reads are available on the SRA (SRR37271106).
